# Comparison of Four Risk of Malignancy Indices for Preoperative Evaluation of Ovarian Masses: A Prospective Observational Study

**DOI:** 10.7759/cureus.41539

**Published:** 2023-07-07

**Authors:** Matcha B Priyanka, Jyochnamayi Panda, Subhra Samantroy, Soumya R Panda, Pramila Jena

**Affiliations:** 1 Obstetrics and Gynaecology, Kalinga Institute of Medical Sciences, Bhubaneswar, IND

**Keywords:** ca-125, ovarian malignancy, ovarian mass, risk of malignancy index, rmi

## Abstract

Background: Ovarian cancer imposes a significant health burden worldwide. Although various tumor markers are available to diagnose ovarian cancer, low-resource countries like India require a humble marker or index. The Risk of Malignancy Index (RMI) has been found to be a simple yet promising tool that can be used for this purpose. In this study, we attempted to validate various RMIs with the help of menopausal status, ultrasonogram score, cancer antigen (CA) 125 value and compare all four RMIs, which would be useful to differentiate benign and malignant ovarian masses. This could be an essential tool, especially in low-resource settings.

Method: This prospective study was conducted at Kalinga Institute of Medical Sciences in Odisha, India, from September 2020 to September 2022 involving 191 patients with ovarian mass with histopathology, which was deemed the “gold standard” diagnostic tool. The sensitivity, specificity, positive predictive value (PPV), and negative predictive value (NPV) of RMI 1, 2, 3, and 4 were calculated and compared.

Results: Out of 191 patients, 32 (16%) had malignancy and 159 (83.2%) had benign pathology. It was apparent that RMI 4 was a better tool for the initial assessment of patients with ovarian masses with a sensitivity of 80.6%, specificity of 96.2%, PPV of 81%, NPV of 96% at a cutoff of 334, and an area under the curve value of 0.939.

Conclusion: RMI 4 followed by RMI 3 were relatively better indices than RMI 1 and RMI 2 for identifying benign and malignant ovarian masses. RMI 4 was a valuable and applicable method in diagnosing pelvic masses with a high risk of malignancy.

## Introduction

Ovarian cancer is the most lethal of all gynecological malignancies worldwide. In India, ovarian cancer is the third most common malignancy among females and comprises about 7.4% of cancer cases [1]. Because of the associated vague symptomatology, ovarian cancers are difficult to diagnose at an early stage. Further, it is difficult to diagnose an ovarian tumor with single parameters such as clinical examination, blood investigations, or imaging. Gynecologists play an important role in identifying, differentiating, and conducting different tests to re-chart a diagnosis. It also depends on the experience of the doctor.

Over the past three decades, several diagnostic algorithms and multimodal investigations have been developed and recommended for use in practice to assist in the differentiation of ovarian tumors. In 1990, Jacob et al. proposed a scoring system called the Risk of Malignancy Index (RMI) [2]. The simple scoring system included scores that express the risk of malignancy such as cancer antigen (CA) 125, menopausal status, and ultrasound score. It is a group or combination of different clinical features that help to improve diagnostic accuracy for ovarian cancer. It is helpful for the selective referral of particular patients to specific cancer centers. The RMI was identified to be effective in distinguishing benign and malignant ovarian masses at a cutoff level of 200. In due time, RMI 1 was gradually improved and modified to RMI 2 and 3 [3,4]. In 2009, Yamamoto et al. developed RMI 4, which added the score of the tumor size [5].

In this study, we used histopathology as the standard technique to evaluate benign and malignant masses. Most of the results vary according to race, geographical features, genetic makeup, and susceptibility of the person. The aim of this study is to validate the RMI with the help of menopausal status, ultrasonogram score, and CA 125 value and compare all four RMIs in relation to the differentiation of benign and malignant ovarian masses in an eastern Indian population.

## Materials and methods

This prospective study was conducted at Kalinga Institute of Medical Sciences in Bhubaneswar, India, from September 2020 to September 2022, observing 191 participants with ovarian masses and those enlisted for surgery for the same diagnosis. The study was approved by the Institutional Ethics Committee at the Kalinga Institute of Medical Sciences (approval number: KIIT/KIMS/IEC/434/2020). 

While women presenting with ovarian mass and admitted for evaluation and treatment were included in the study, those who had already been diagnosed with ovarian malignancy and received chemotherapy for the same diagnosis were excluded. First, detailed clinical history was taken and clinical examination was done for each participant. Preoperative serum CA 125 levels, ultrasound findings, and menopausal status were also noted for each patient. Second, an ultrasound examination was performed using the GE Voluson 730 PRO ultrasound machine (General Electric Company, Boston, Massachusetts, United States) and a 3.5-MHz abdominal convex transducer (GE Voluson 730 5C-A; General Electric Company) in patients with a full bladder or 7.5-MHz vaginal probe (GE Voluson 730 RRE6-10; General Electric Company) in patients after emptying the bladder. One point was assigned for each of the following ultrasound features: the presence of a multilocular cystic lesion, solid areas, bilateral lesions, ascites, and intra-abdominal metastases. 

Then, a total ultrasound score (U) was calculated for each patient. The tumor size (S), in centimeters, was measured by ultrasound as the maximum diameter of the mass. Postmenopausal status was defined as attaining amenorrhea for at least one year or age greater than 50 years in women who underwent hysterectomies. All other women were considered premenopausal. Meanwhile, preoperative serum CA 125 levels were measured in the hospital’s biochemistry laboratory by radioimmunoassay. According to the preceding data, RMI 1, RMI 2, RMI 3, and RMI 4 were calculated for all patients together. The different parameters (CA 125, U, Menopause status (M), and S) for calculating the different RMIs are given in Table 1. RMI 1, 2, and 3 were calculated as: U × M × CA-125; whereas RMI 4 was calculated as: U × M × S × CA 125.

**Table 1:** Calculation of the four RMIs RMI: Risk of Malignancy Index

SL. No.	Parameters	RMI1	RMI2	RMI3	RMI4
1	CA 125	U/ML	U/ML	U/ML	U/ML
2	Menopausal Status				
	Premenopausal	1	1	1	1
	Menopausal	3	4	3	4
3	Ultrasonogram Score				
	No parameter present	U=0	U=0	U=1	U=1
	1 parameter present	U=1	U=1	U=1	U=1
	2 or more parameters present	U=3	U=4	U=3	U=4
4	Tumor Size Score				
	Size < 7cm	-	-	-	S=1
	Size > 7cm	-	-	-	S=2

The histopathologic diagnosis was regarded as the definite outcome. Results of RMI were validated with histopathological findings. Data with continuous variables were represented as mean ± standard deviation, and data with categorical variables were represented as a frequency percentage. We used the Chi-square test or Fischer exact test to measure the difference between two categorical variables at a two-sided significance level of 0.05. Normally distributed data were analyzed by applying the student’s t-test. To determine the performance of the four RMIs, receiver operating characteristic (ROC) curves were constructed. Sensitivity, specificity, positive predictive value (PPV), negative predictive value (NPV), and area under the curve (AUC) were determined at a particular cutoff level for each RMI. Appropriate statistical analysis was performed at the end of the study by IBM SPSS Statistics for Windows, Version 21.0 (Released 2012; IBM Corp., Armonk, New York, United States). A p-value of < 0.05 was considered to indicate statistical significance.

## Results

The baseline characteristics of the participants are given in Table 2. Out of 191 patients, 32 (16%) patients had malignancy, and 159 (83.2 %) patients had benign pathology. The mean age of patients presenting with ovarian mass was between 35.7 ± 11.89 years’ ages. Most of the participants belonged to a lower middle socioeconomic status. The majority of the women (80.6%) were multiparous. A total of 42.8% (12 out of 28) of malignancies occurred in postmenopausal women and 12.6% (20 out of 143) in premenopausal women. Out of 191 participants, CA 125 was less than 35 u/ml in 136 participants, whereas 55 participants had CA 125 more than or equal to 35 u/ml. Among these, three out of 136 were malignant with CA 125 < 35 u/ml, and 26 out of 28 were malignant with CA 125 values > 35 u/ml (Table 2). The ultrasound distribution of benign and malignant ovarian tumors is shown in Table 2.

**Table 2:** Baseline characteristics BMI: Body mass index; CA: cancer antigen

Sl no.	Baseline characteristics	All participants	Benign	Malignant	P value
1.	Age (years)	35.7 ± 11.89	34.62±11.1	41.15± 14.02	0.0047
2.	BMI (Kg/m^2^)	26.09 ± 2.44	26.09 ± 2.39	26.24 ± 2.76	0.765
3.	Socioeconomic status				
	Upper class	30	22 (13.8%)	8 (25.8%)	0.387
	Upper middle class	27	25 (15.7%)	2 (6.5%)	
	Lower middle class	56	47 (29.6%)	9 (29.0%)	
	Upper lower class	44	37 (23.3%)	7 (19.4%)	
	Lower class	34	28 (17.6%)	6 (19.4%)	
4.	Parity				
	Multiparous	154 (81%)	128 (83%)	26 (17%)	1.0495
	Nulliparous	37 (19%)	31 (83%)	6 (17%)	
5.	Menopausal status				
	Postmenopausal	28 (15%)	16 (57.2%)	12 (42.8%)	0.0049
	Premenopausal	163 (85%)	143 (87.4%)	20 (12.6%)	
6.	CA 125				
	< 35	136 (71%)	133 (98%)	3 (2%)	
	≥35	55 (29%)	23 (41%)	32 (59%)	
7.	Sonographic morphology				
	Bilaterality	31(16%)	28 (90%)	3 (10%)	0.0548
	Multilocularity	52 (27%)	29 (56%)	23 (44%)	<0.001
	Solid areas	62 (33%)	38 (73%)	24 (27%)	<0.001
	Ascites	15 (7%)	1 (7%)	14 (93%)	<0.001
	Metastasis	7 (3%)	1 (1%)	6 (92%)	<0.001

**Figure 1:**
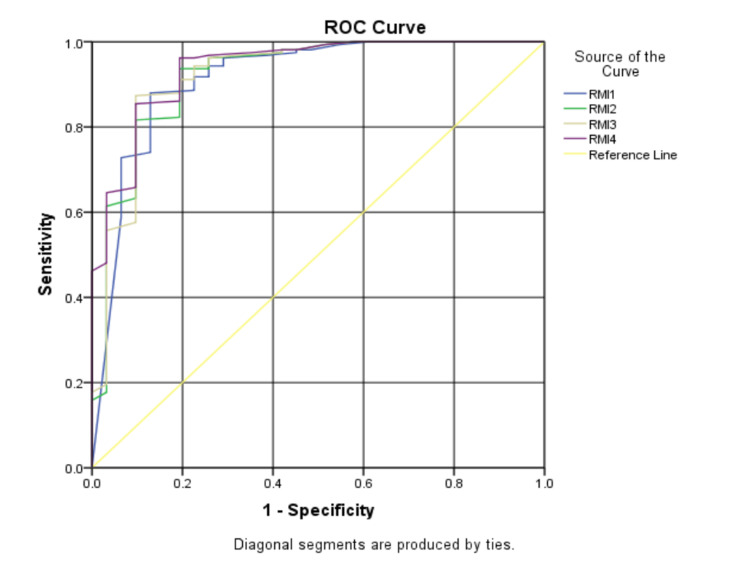
ROC curve showing the performances of all the four RMIs ROC: receiver operating characteristic; RMI: Risk of Malignancy Index

The performances of RMI 1, RMI 2, RMI 3, and RMI 4 are presented in the ROC curve (Figure 1). RMI 4 has the highest AUC of 0.939, with a sensitivity of 80.6%, specificity of 96.2%, PPV of 81%, and NPV of 96% at a cutoff of 334, followed by RMI 3 with an AUC of 0.923, a sensitivity of 80.6%, specificity of 96.2%, PPV of 81%, and NPV of 96% at a cutoff of 83). RMI 2 has an AUC of 0.920 with a sensitivity of 80.6%, specificity of 93.7%, PPV of 71%, and NPV of 96% at a cutoff of 165 (Table 3).

**Table 3:** The performance of RMI 1, RMI 2, RMI 3, and RMI 4 RMI: Risk of Malignancy Index; AUC: area under the curve; PPV: positive predictive value; NPV: negative predictive value.

Test Result Variable(s)	Cut-off	Sensitivity	Specificity	AUC	p-value	PPV	NPV
RM1	84	0.871	0.880	0.915	<0.001	0.59	0.97
RMI2	165	0.806	0.937	0.920	<0.001	0.71	0.96
RMI3	86	0.903	0.873	0.923	<0.001	0.58	0.98
RMI4	334	0.806	0.962	0.939	<0.001	0.81	0.96

All four RMIs were found to have a positive correlation with the histopathological examination (HPE) report (Table 4).

**Table 4:** Correlation of all four RMIs with HPE RMI: Risk of Malignancy Index; HPE: histopathological examination

Indices	Correlation with HPE	
RMI 1	Correlation	0.561	
	Value	< 0.001	
RMI 2	Correlation	0.545	
	P-value	< 0.001	
RMI 3	Correlation	0.544	
	P-value	< 0.001	
RMI 4	Correlation	0.569	
	P-value	< 0.001	

## Discussion

In this study, we found that the evaluation of ovarian masses through RMI is effective and has certain advantages. This fact holds particularly true in low-resource settings. RMI helps in the rapid triage of women with ovarian masses and thus paves the way for early referral to oncology units. Hence, it helps in reducing the performance of suboptimal surgeries at local or peripheral hospitals. As shown in Table 2, the mean age of study participants was 35 ± 11 years, which was similar to that found in several other studies [6-8]. The mean age of the benign group was 34.6 ± 11 years, whereas the mean age of the malignant group was 41.15 ± 14.2 years (Table 2). However, studies by Ashrafgangooei et al. [9] and Simsek et al. [10] found the mean age in benign versus malignant groups to be 37.0 ± 8.79 versus 35.23 ± 10.87 and 50.8 ± 12.9 versus 50.78 ± 13.39, respectively. 

A total of 42.8% (12 out of 28) of malignancies occurred in postmenopausal women and 12.6% (20 out of 143) among premenopausal women (Table 3). Similar incidences were found in postmenopausal patients by Rao et al. [11] and Kumari et al. [12]. Although serum CA 125 is routinely used as a tumor marker for ovarian cancer, the levels may increase in some other gynecological pathologies. Simsek et al. reported a sensitivity of 78.6% and specificity of 63.5% for CA 125 at a cutoff value of 35 u/ml [10]. In a recent study, Singhal et al. found a sensitivity of 75% and specificity of 90% for CA 125 levels > 35 u/ml [13]. Meanwhile, in this study, three out of 136 patients were malignant with CA 125 < 35 u/ml and 28 out of 55 were malignant with CA 125 > 35 u/ml (Table 2). 

We found that the performance of RMI 4 was relatively better for triage of ovarian masses than RMI 1, 2, and 3 (Table 3). At a cutoff value of 165, the specificity and PPV of RMI 2 were better than that of RMI 1 (at a cutoff of 84) and RMI 3 (at a cutoff of 86) with low specificity (Table 3). The finding supports the previous report of Yamamoto et al., who reported that at a cutoff level of 450, RMI 4 had better performance than RMI 1, 2, and 3 [5]. They reported sensitivity, specificity, PPV, and NPV as 86.8%, 91.0%, 63.5%, and 97.5%, respectively for RMI 4. Other researchers also reported RMI 4 to be more sensitive than RMI 3 (68.9 % versus 62%) and RMI 4 also as more specific than RMI 2 (81% versus 78%) [14,15]. In contrast, RMI 3 has been reported as more specific than RMI 4 (94 %versus 92%) [14]. 

When a comparison was made between RMI 1 with RMI 2, studies have reported that RMI 2 is more reliable for triage. According to Tingulstad et al., at a cutoff level of 200, RMI 2 was significantly better than RMI 1 [3]. A similar result was shown by Morgante et al. [16], where they reported that for cutoff values between 80 and 250, the performance of RMI 2 was better than RMI 1 and was statistically significant. Some researchers found similar performances of RMI 1 and 2 at the cutoff value of 200 and better prediction of malignancy than RMI 3 [17]. Previous data from Nepal showed a sensitivity of 85% for both RMI 1 and 2 at a cutoff level of 200, but specificity was improved with RMI 2 (94% versus 88%) [18]. In this study, a sensitivity of RMI 1 at a cutoff of 84 was better than RMI 2 at a cutoff of 165 (87% versus 80.6%) but was limited by low PPV (59%) (Table 3). The sensitivity (90.3%) and specificity (87.3 %) of RMI 3 at a cutoff of 86 in this study (Table 3) were better than that proposed by Tingulstad et al., according to whom RMI 3 had a sensitivity and specificity of 71% and 92%, respectively, at a cutoff level of 200 [4]. However, in a study by Dora et al., the cutoff value of 236 had a high sensitivity (72.5%) and specificity (98.25%) with a PPV of 98.1% at a cutoff value of 200 [8]. 

Some studies revealed no significant differences among the different forms of RMI. According to Aktürk et al., any form of RMI can be used for the evaluation of ovarian mass [19]. Manjunath et al. showed a similar result [20]. According to Clara et al., RMI is not appropriate for triage for the Southeast Asian population [21]. In this study, NPVs are high for all forms of RMI (Table 3). The NPV for RMI 1 was 97% at a cutoff of 84, whereas for RMI 2, it was found to be 96% at a cutoff of 165. This is similar to that reported by Aktürk et al. [19]. In a review by Geomini et al., the RMI cutoffs ranged from 25-250 [22]. Although RMI 4 at a cutoff point of 334 provided the best performance in this current study, no major difference was observed from the performances of other RMIs, especially against RMI 3. Thus, according to this study, RMI was found to be better than any other single parameter for diagnosing and triaging ovarian masses.

Certain limitations should be considered while interpreting the results of this study. First, this is a single-centered study. Second, the overall sample size was relatively small. Hence, the study results may not be generalized enough to be applicable to a large population. However, because this study was conducted in a tertiary care center that caters to a large group of patients from the eastern part of India, the results of this study are expected to be applicable in the said region.

## Conclusions

RMI is a simple scoring method that can be routinely adopted because of its reliability and cost-effectiveness, especially in situations with limited resources. RMI, as opposed to other parameters, should be adopted for triaging ovarian masses. In this study, RMI 4 followed by RMI 3 at cutoff values of 334 and 86, respectively, are the better indices than RMI 1 and RMI 2 for triaging ovarian tumors and subsequent referral to gynecologic oncologists. Hence, we recommend using RMI 4 for this purpose, because it is a valuable and applicable method in diagnosing pelvic masses with high risk of malignancy. However, caution should be exercised in certain circumstances where CA 125 levels are not expected to rise prominently like mucinous carcinomas. Further, high-quality research is needed involving large heterogenous groups of populations to understand the broader application of RMIs.
